# Transformation magneto-statics and illusions for magnets

**DOI:** 10.1038/srep06593

**Published:** 2014-10-13

**Authors:** Fei Sun, Sailing He

**Affiliations:** 1 Centre for Optical and Electromagnetic Research, Zhejiang Provincial Key Laboratory for Sensing Technologies, JORCEP, East Building #5, Zijingang Campus, Zhejiang University, Hangzhou 310058, China; 2 Department of Electromagnetic Engineering, School of Electrical Engineering, Royal Institute of Technology (KTH), S-100 44 Stockholm, Sweden

## Abstract

Based on the form-invariant of Maxwell's equations under coordinate transformations, we extend the theory of transformation optics to transformation magneto-statics, which can design magnets through coordinate transformations. Some novel DC magnetic field illusions created by magnets (e.g. rescaling magnets, cancelling magnets and overlapping magnets) are designed and verified by numerical simulations. Our research will open a new door to designing magnets and controlling DC magnetic fields.

Transformation optics (TO), which has been utilized to control the path of electromagnetic waves^1^,^2^,^3^,^4^,^5^,^6^,^7^, the conduction of current^8^,^9^, and the distribution of DC electric or magnetic field^10^,^11^,^12^,^13^,^14^,^15^,^16^,^17^,^18^,^19^,^20^ in an unprecedented way, has become a very popular research topic in recent years. Based on the form-invariant of Maxwell's equation under coordinate transformations, special media (known as transformed media) with pre-designed functionality have been designed by using coordinate transformations^1^,^2^,^3^,^4^. TO can also be used for designing novel plasmonic nanostructures with broadband response and super-focusing ability^21^,^22^,^23^. By analogy to Maxwell's equations, the form-invariant of governing equations of other fields (e.g. the acoustic field and the thermal field) have been studied under coordinate transformations, and many novel devices have been designed that can control the acoustic wave^24^,^25^ or the thermal field^26^,^27^,^28^,^29^,^30^,^31^.

The current theory of TO can be directly utilized to design some passive magnetic media (with transformed permeability) to control the distribution of the DC magnetic field: a DC magnetic cloak that can hide any objects from being detected from the external DC magnetic field^17^,^18^,^19^, a DC magnetic concentrator that can achieve an enhanced DC magnetic field with high uniformity^10^,^11^,^12^,^13^,^14^, a DC magnetic lens that can both amplify the background DC magnetic field and the gradient of the field^15^,^16^, and a half-space carpet DC magnetic cloak^20^. However there has been no study on transforming magnets by using TO, and no literature describes how the residual induction (or intensity of magnetization) of a magnet transforms if there is a magnet in the reference space.

In this paper we extend the current TO to the case involving the transforming of the residual induction of a magnet. Based on the proposed theory, we design three novel devices that can create the illusion of magnets: the first one rescales the magnet (e.g. a small magnet can perform like a bigger one); the second nullifies the magnet (e.g. we can cancel the DC magnetic field produced by a magnet by adding special anti-magnets and transformed materials); and the third overlaps magnets (e.g. we can overlap magnets in different spatial positions to perform effectively like one magnet with overlapped residual induction). The study in this paper will lead a new way to design magnets and create the illusion of a DC magnetic field.

## Methods

In the section, we will extend the theory of TO to transform the magnetization intensity of magnets. Our starting point is two magnetic field equations in Maxwell's equations. In the reference space of a Cartesian coordinate system, we can write them as^4^: 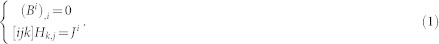
The comma refers to partial differentiation. [ijk] is the permutation symbol, which is the same as the Levi-Civita tensor in the right-hand Cartesian coordinate system. Due to the form-invariant of Maxwell's equations (any physics equation expressed in tensor form is form-invariant under coordinate transformations), we can rewrite Eq. (1) in a curved coordinate space as^4^: 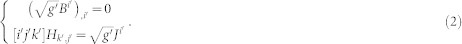
where *g*' = det(*g_i'j'_*). *g_i'j'_* is metric tensor in this curved coordinate system. The key point of TO is material interpretation^1^,^4^: we can assume that the electromagnetic medium and the curved coordinates have an equivalent effect on the electromagnetic wave, and treat Eq. (2) as Maxwell's equations in a Cartesian coordinate system of a flat space but not in a curved coordinate space. We make the following definitions: 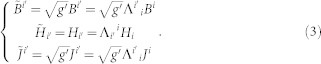
The quantities with superscript tilde ‘~' and primes indicate that the quantities are in the real/physical space; the quantities without primes indicate the ones in the reference space (a virtual space), and the quantities with primes and without the superscript ‘~' are ones in the transition space (a curved space). In the real space, the space is flat while filled with some special medium (the transformed medium), which will be deduced later. In order to express quantities conveniently, we often drop the superscript ‘~' in the real space, and rewrite Eq. (3) as: 
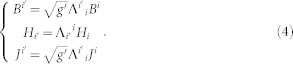
Here the quantities with or without primes indicate the ones in the real or reference space, respectively. Since the reference space is in the Cartesian coordinate system (which means *g* = 1), the metric tensor in Eq. (4) can be rewritten as: 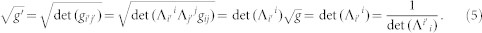
By using Eq. (5), we can rewrite Eq. (4) as: 
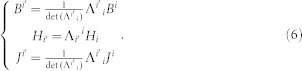
Note Eq. (6) can also be deduced by other ways (e.g. multivariable calculus^6^). In traditional TO, we often assume that the medium in the reference space does not contain any magnets and this means the residual induction ***B****_r_* (or intensity of magnetization ***M*** = ***B****_r_*/*μ_0_*) is zero everywhere. In this case, we have: 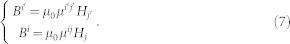
Combining Eq. (6) and (7), we can obtain the relationship between the medium in the real space and the reference space: 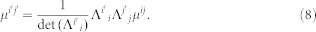
Eq. (8) is a classical equation in TO. If there are some magnets in the reference space, then Eq. (7) should be modified as: 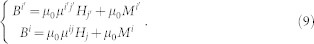
Here ***M*** and ***M'*** correspond to the magnetization intensity of magnets in the reference and real space, respectively. In this case, we can combine Eq. (6) and (9) to obtain the following relation: 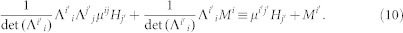
Considering that Eq. (10) is true for any magnetic field *H*', we can obtain: 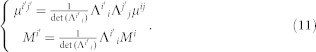
Eq. (11) gives the complete transformation of the relation between the magnetic materials in the reference space and the transformed magnetic materials in the real space, even if there are some magnets in the reference space. As we can see from Eq. (11), if there is no magnet in the reference space ***M*** = 0, we have ***M'*** = 0. In this case, only permeability needs to be transformed (Eq. (11) reduces to Eq. (8)), which is consistent with classical TO. We can rewrite Eq. (11) in a matrix form: 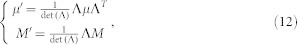
where 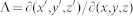 is the Jacobian transformation matrix. Now we have extended the classical TO to the case in which there are magnets in the reference space. In the next section, we will use this theory to design some novel devices which can create illusions of magnets.

## Results

### 1) Rescaling magnets

**Figure 1:**
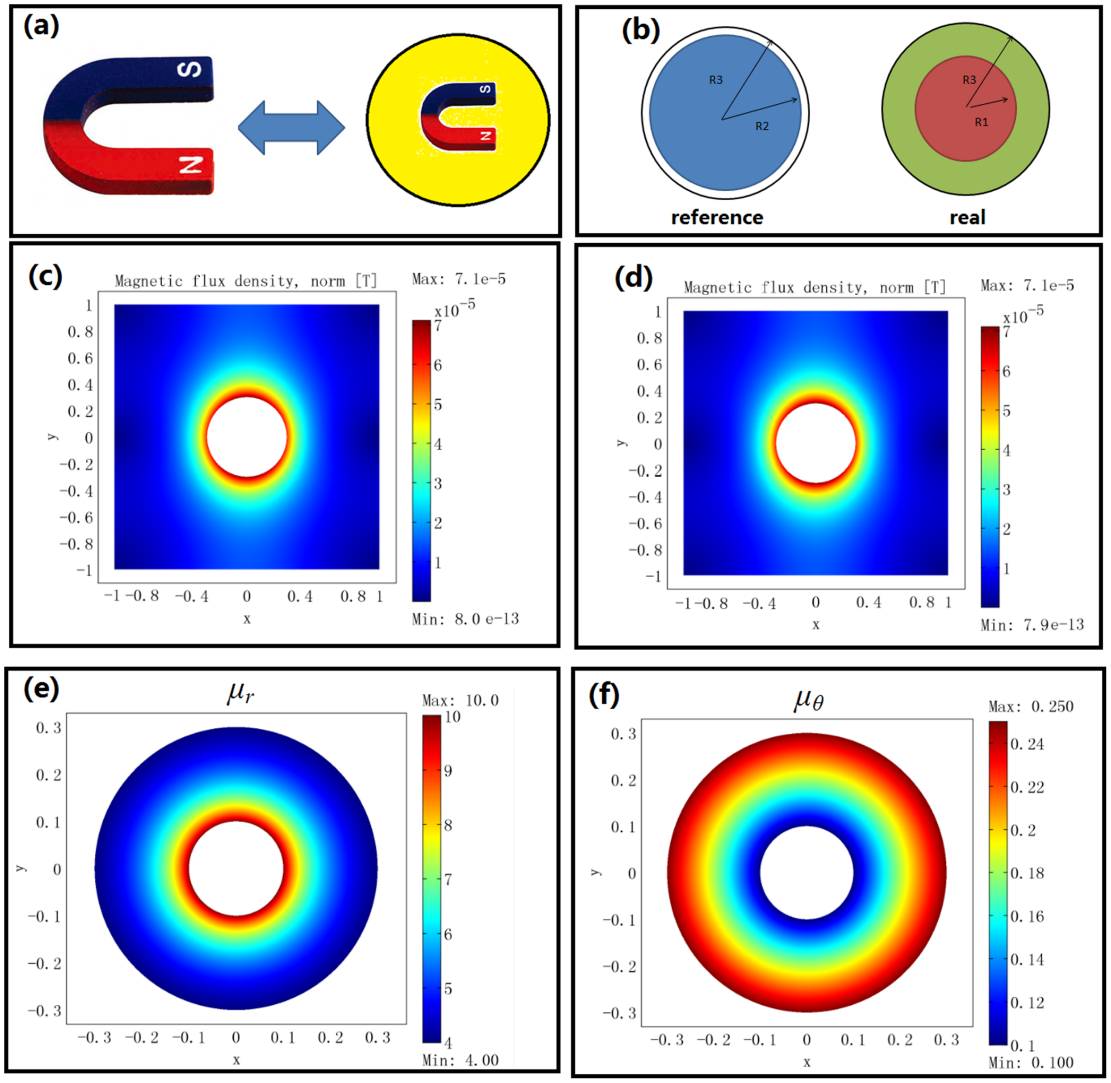
(a) The basic idea of this rescaling magnet illusion is that we can use a small magnet and a transformed medium to cause the whole structure to perform like a big magnet. (b) The transformation relation for a 2D cylindrical magnet. The left part is the reference space: the blue circle is the big magnet with radius *R*_2_ filled in air (white region). The right part is the real space: the red circle is the small magnet with radius *R*_1_, the green region is the rescaling medium (*R*_1_ < *r'* < *R*_3_ given in Eq. (19)), and the remaining white region is air. (c) FEM simulation result: the magnetic flux distribution in the region *r'* > *R*_3_ = 0.3 m produced by a single magnet with radius *R*_2_ = 0.25 m, relative permeability *μ_m_*_0_ = 1000 and a residual induction of 0.1 T along the *x*-direction. (d) FEM simulation result: the magnetic flux distribution in the region *r'* > *R*_3_ = 0.3 m produced by a magnet with radius *R*_1_ = 0.1 m, same relative permeability *μ_m_*_0_ = 1000 and a residual induction of 0.25 T (determined by Eq. (18)) along the *x*-direction and rescaling medium described by Eq. (19) in the region *R*_1_ < *r'* < *R*_3_. (e) The distribution of relative permeability along the radial direction for the rescaling medium in (d). (f) The distribution of relative permeability along the tangential direction for the rescaling medium in (d).

The first example is a device that can amplify the volume of a magnet. Fig. 1 (a) shows the basic idea of this illusion. For simplicity, we consider a 2D circular magnet with radius *R*_2_ filled in the free space (infinitely long in the *z*-direction; see the left part of Fig. 1(b)). The relative permeability and magnetization intensity in the reference space can be given, respectively, as: 
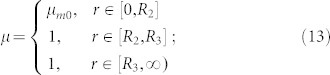
and 
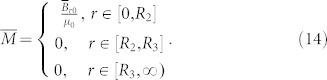
***B***_r0_ is the residual induction in the reference space. *μ_m_*_0_ is the relative permeability of the magnet in the reference space. Now we want to use a magnet (of the same *μ_m_*_0_) with a smaller radius *R*_1_ (*R*_1_ < *R*_2_) in the real space to produce the same DC magnetic field in the region *r'* > *R*_3_ (as if it is produced by a big magnet with radius *R*_2_ in the reference space). The reference space is shown in the left part of Fig. 1(b). The permeability and magnetization distributions are given in Eq. (13) and (14). The real space is shown in the right part of Fig. 1(b). The magnet in the region 0 < *r* < *R*_2_ in the reference space is compressed to the region 0 < *r'* < *R*_1_ < *R*_2_ in the real space. The air region *R*_2_ < *r* < *R*_3_ in the reference space is expanded to the region *R*_1_ < *r* < *R*_3_ in the real space while keeping the air region *r* > *R*_3_ in the reference space identical to the region *r'* > *R*_3_ in the real space. The full transformation can be given as: 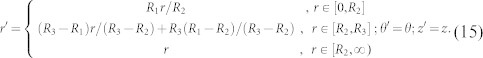
Combing Eq. (11) and (15), we can obtain the relative permeability and magnetization intensity in the real space: 
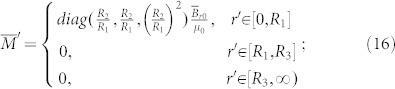

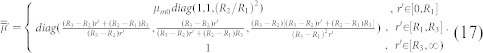


The above quantities are expressed in the cylindrical coordinate system. For the 2D case (the magnetic field is in the plane *z* = 0 and ***B***_r0_ does not contain any z-component), we can just drop the *z*-component in the above expression, and they will reduce to: 
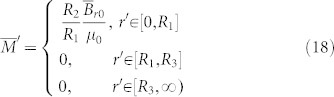
and 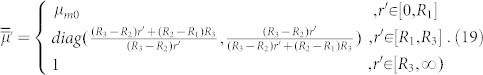
Note that the quantities in Eq. (18) and (19) are expressed in a 2D cylindrical coordinate system. As we can see from Eq. (18), if we want a magnet with small radius *R*_1_ to produce the same DC magnetic field distribution as a magnet (of the same relative permeability) with a larger radius *R*_2_, its magnetization intensity should be rescaled accordingly. We use the finite element method (FEM) to verify the performance of the device (see Fig. 1(c) and (d)). The distributions of the DC magnetic field outside the region *r'* > *R*_3_ are exactly the same for the case where we only have a magnet with big radius *R*_2_ (see Fig. 1(c); permeability and magnetization distributions are given in Eq. (13) and (14), respectively) and the case where we have a magnet (of same relative permeability) with small radius *R*_1_ and some rescaling medium (see Fig. 1(d); permeability and magnetization distributions are given in Eq. (18) and (19), respectively). The FEM simulation is performed by using a commercial software, COMSOL Multiphysics. The distribution of the relative permeability of the scaling medium (*R*_1_ < *r'* < *R*_3_) in Eq. (19) is shown in Fig. 1(e) and (f): the permeabilities along both the radial and tangential directions are larger than zero. We can first divide the device into many layers and treat the permeability in each layer as a homogeneous and anisotropic one. Then we can use alternated layers of ferromagnetic materials with permeability larger than 1 and DC meta-materials with permeability between 0 and 1^19^ to realize the required homogeneous anisotropic permeability in each layer. This is similar to the method of creating a magnetic concentrator for DC magnetic field enhancement^14^.

Although two magnets have the same relative permeability for a 2D case (see Eq. (13) and (19)), magnetization intensities of two magnets are different in two cases (see Eq. (14) and (18)). The small magnet with radius *R*_1_ has a bigger magnetization intensity *R*_2_*B*_0_/(*R*_1_*μ*_0_) and the big magnet with radius *R*_2_ has a small magnetization intensity *B*_0_/*μ*_0_. The magnetic energy is conserved in each system. We should also note for a 3D case, two magnets will have different relative permeabilities (see Eq. (17)) and the magnetization intensity in the real space will be anisotropic (see Eq. (16)).

### 2) Cancelling magnets

**Figure 2:**
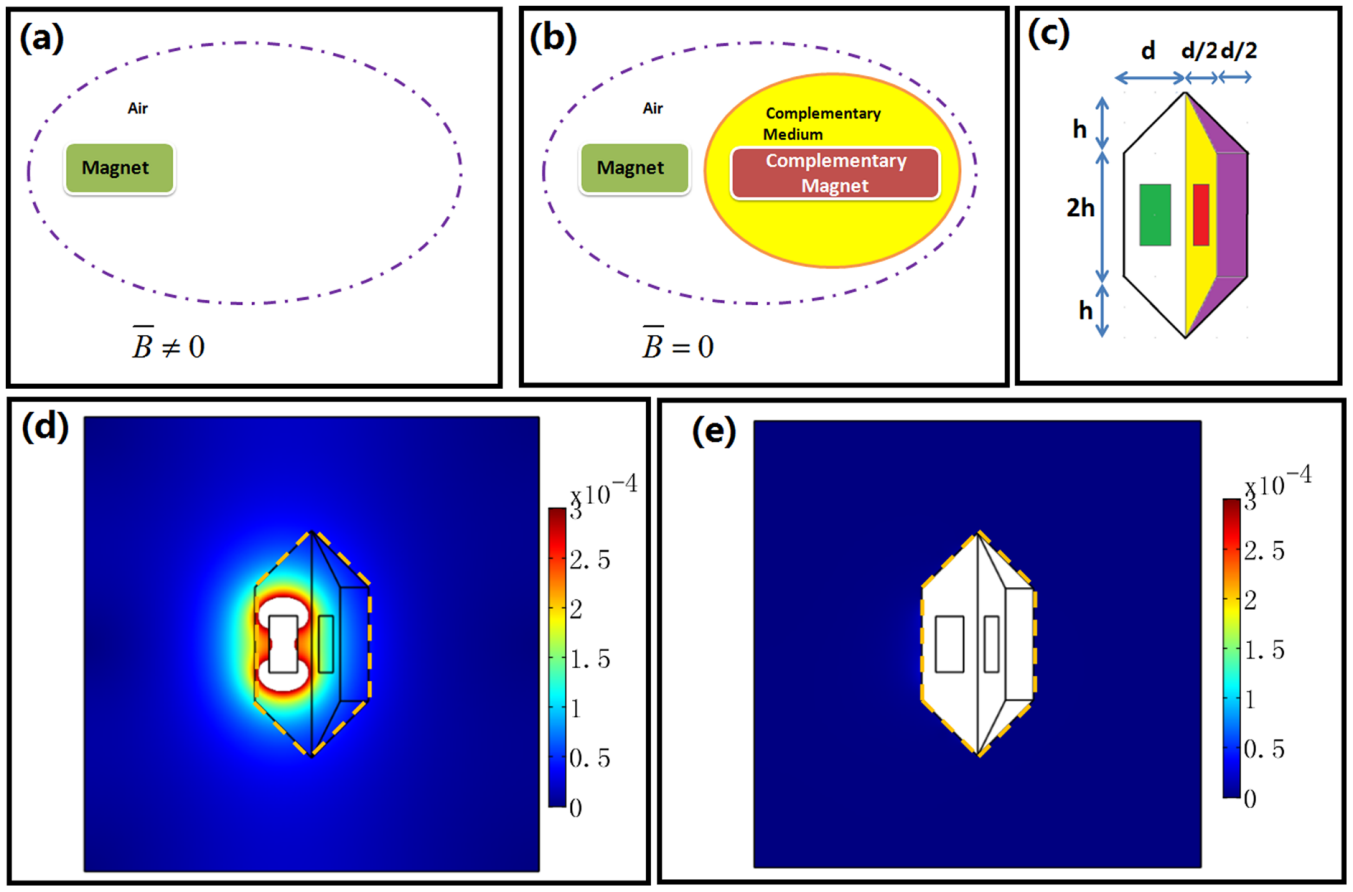
(a) and (b): The basic idea of cancelling magnets by using complementary magnets and a complementary medium. (a) One magnet can produce a non-zero DC magnetic field in a certain region of space (e.g. the region outside the dashed purple line). (b) If we put a complementary magnet and complementary medium aside the original magnet, the DC magnetic field in the same region (e.g. the region outside the dashed purple line) becomes nearly zero. (c) A specific structure to illustrate our idea of a cancelling magnet: the green region is the magnet with relative permeability 1000, residual induction 1 T along the *x*-direction, width 0.1 m and height 0.2 m; the red region is the complementary magnet with width 0.05 m and height 0.2 m; the yellow region is the complementary medium; and the purple region is the restoring medium. (d) and (e): the FEM simulation results. (d) The amplitude of the total DC magnetic flux density distribution outside the whole structure when only one magnet (green part in (c)) is used. (e) The amplitude of the total DC magnetic flux density distribution outside the whole structure when the original magnet (green region), complementary magnet (red region), complementary medium (yellow region) and restoring medium (purple region) are all used.

Analogous to the scattering-cancelling by using a complementary medium in the electromagnetic wave case^3^, we can design complementary magnets and complementary media to cancel the DC magnetic field produced by some magnets (see Fig. 2(a) and (b)). We design a specific structure shown in Fig. 2(c) with *h* = *d* = 0.2 m. The coordinate transformation is given as the following.

The yellow and red regions: 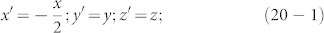
The purple region: 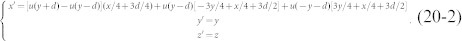
And the other white region and green region: 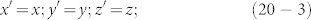
where function *u* is defined as follows: 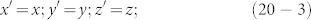
Combining Eq. (20) and Eq. (11), we can obtain the material in:

the yellow region (complementary medium): 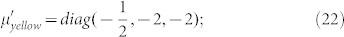
the red region (the complementary magnet): 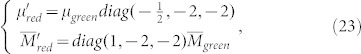
where *μ*_green_ and ***M***_green_ are the relative permeability and magnetization intensity of the green magnet, respectively;

and the purple region (restoring medium): 
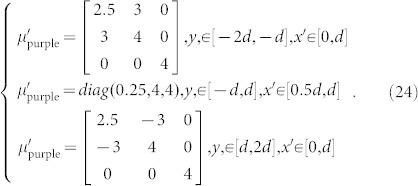
The FEM simulation results are shown in Fig. 2(d) and (e): if we only put one magnet (the green part in Fig. 2(c)), the DC magnetic field is non-zero outside the white region in Fig. 2(d). However if we add some complementary magnet (red region), complementary medium (yellow region), and restoring medium (purple region) to the original magnet (green region), the DC magnetic field can be greatly reduced outside the whole structure (see Fig. 2(e)).

We note that people can shield the DC magnetic field around a magnet simply by adding a magnetic insulation layer (e.g. a superconductor shell). In this case, the region without the magnetic field and the region with the magnetic field are completely isolated. Our method is different: we do not need to form a closed region by using a magnetic insulation shell, but instead we put a complementary magnet and complementary medium aside the magnet. Therefore, the magnet region is connected with the outside region (e.g. other objects can be moved freely between the region without the magnetic field and the region with the magnetic field).

### 3) Overlapped magnets

**Figure 3:**
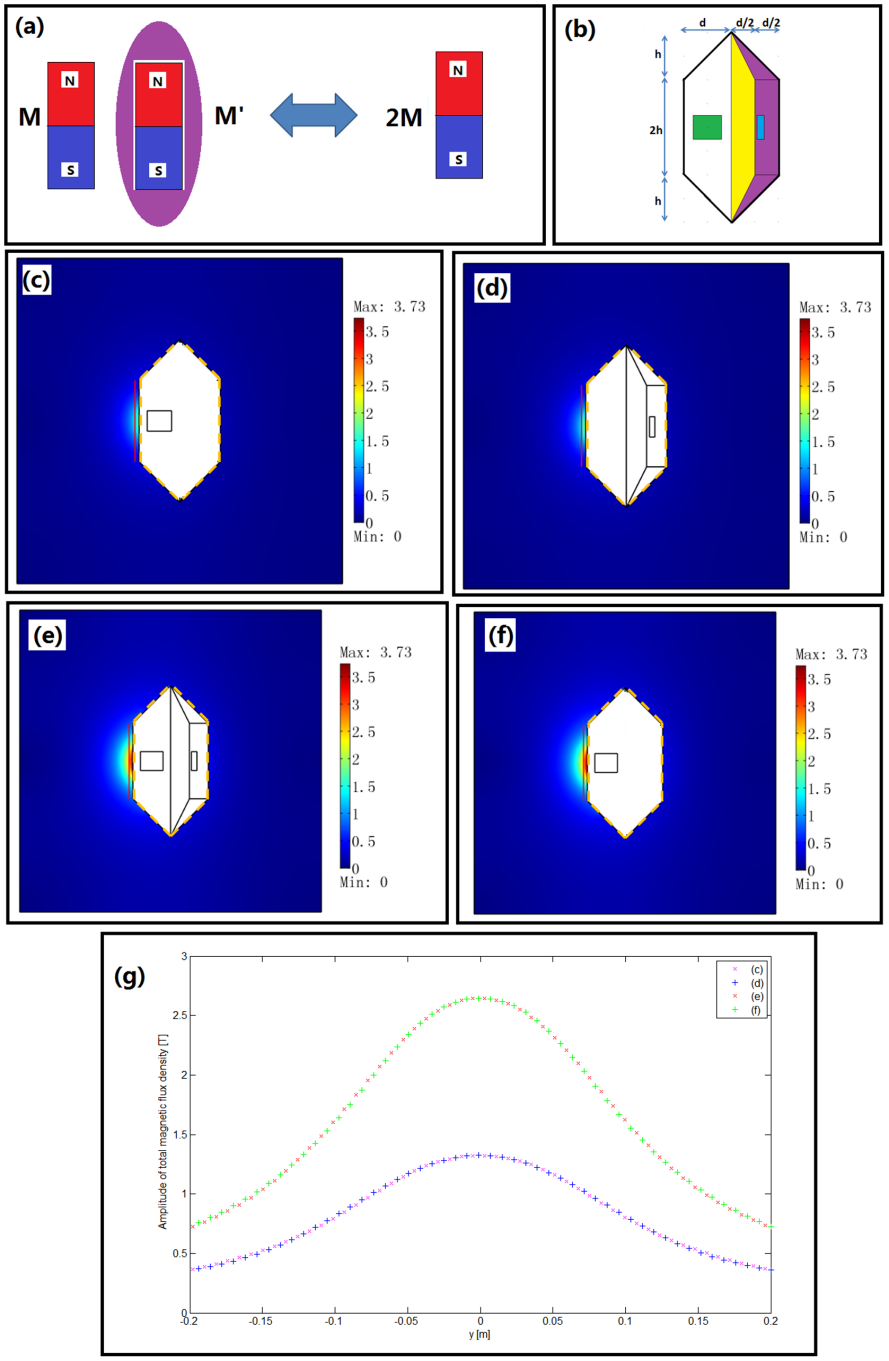
(a) The basic idea of overlapped magnets: two magnets at different locations with some transformed medium can mimic one magnet of higher magnetization intensity for outside observers. (b) A specific structure: the green region is one magnet with width 0.12 m and height 0.1 m; the blue region is another magnet with width 0.03 m and height 0.1 m; the yellow region is the complementary medium; and the purple region is the restoring medium. (c) The amplitude of the total DC magnetic flux density distribution outside the whole structure when only the green magnet with relative permeability *μ*_green_ = 1 and residual induction 10 T along the *x*-direction is used. Note that in this case we do not introduce the blue magnet and transformed media in the yellow and purple regions (they are all set as air). (d) The amplitude of the total DC magnetic flux density distribution outside the whole structure with only the blue magnet with the relative permeability and residual induction calculated from Eq. (25) by choosing *μ*_green_ = 1 and ***M***_green_ = diag(10 T,0,0)/*μ*_0_. The transformed media in the yellow and purple regions are introduced. Note that in this case we do not introduce the green magnet. (e) The amplitude of the total DC magnetic flux density distribution outside the whole structure when both green and blue magnets are introduced (the relative permeability and the residual induction of these magnets are the same as those in (c) and (d)). We also introduce transformed media in the yellow and purple regions. (f) The amplitude of the total DC magnetic flux density distribution outside the whole structure when only the green magnet with double residual induction in (c) is introduced. In this case, yellow, purple, and blue regions are set as air. (g) The amplitude of the total DC magnetic flux density distribution along the same red line indicated in (c) to (f).

Inspired by overlapped illusions of light sources^5^, we can also create an illusion of overlapped magnets. The basic idea is shown in Fig. 3 (a): two magnets at different locations can perform like one magnet with overlapped magnetization intensity to the outside observer. To create such an illusion, we need to transform both the permeability and magnetization intensity, which require the extended TO in this paper (Eq. (11)).

The structure we chose here is similar to the second example for cancelling magnets (see Fig. 3(b)): we use the same coordinate transformation Eq. (20) for each region (the only difference is that we remove the complementary magnet in the red region in Fig. 2(b) and add another magnet in the blue region in Fig. 3(b)). The coordinate transformation in the blue region (filled with a magnet) is given in Eq. (20-2). We still choose *h* = *d* = 0.2 m., so the materials in the yellow and purple regions are still described by Eq. (22) and (24), respectively. The material in the blue region can be given as: 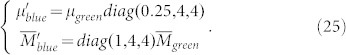
In this case, the blue magnet, whose permeability and magnetization can be determined by Eq. (25) together with purple and yellow media (whose permeabilities are given in Eq. (22) and (24)), will produce the same DC magnetic field as that produced by only one green magnet with permeability *μ*_green_ and magnetization ***M***_green_ in the external space. The simulation results are given in Fig. 3(c) to (f). As we can see from Fig. 3(c) and (d), the distributions of amplitude of DC magnetic flux are the same for the case where only one green magnet is used and the case where only one blue magnet (the residual inductions are both 10 T along the *x*-direction) with transformed media in the yellow and purple regions introduced. Fig. 3(e) and (f) show that if we introduce both the green magnet and blue magnet with a residual induction of 10 T along the x-direction, and a transformed medium in the yellow and purple regions, the distribution of amplitude of DC magnetic flux outside the whole structure is the same as when we only introduce a green magnet with a residual induction of 20 T along the *x*-direction. Fig. 3(g) also reveals that the magnetic flux density distributions in (c) and (d) are exactly the same, and the magnetic flux density distributions in (e) and (f) are exactly the same.

This overlapped illusion of magnets may have important practical applications, e.g., to achieve an extremely enhanced DC magnetic field by overlapping many magnets. The highest DC magnetic field produced by a single magnet is limited by many factors (e.g. the highest current in the coil, heat effects, etc.). We can put many magnets in different locations, combined with the above transformed medium to overlap these magnets together, in order to form one effective strong magnet that can hardly be achieved by traditional methods.

The illusion of cancelling magnets and overlapped magnets need materials with negative permeability. It is not easy to realize negative permeability for the DC magnetic field with current meta-materials. A meta-material based on a stacked fishnet structure shows a broadband negative permeability starting at zero frequency may give a possible solution to the realization of negative permeability for the DC magnetic field^32^.

## Summary

We extend the theory of transformation optics to include the transformation of magnets. The proposed theory will usher in a new way to design magnets: a coordinate transformation method. By using coordinate transformations, we can create many illusions of magnets, such as rescaling magnets, cancelling magnets, and overlapping magnets. These novel devices, which can manipulate the DC magnetic field produced by magnets, will have many potential applications (e.g. magnetic field shielding, magnetic sensors, etc.).

## Author Contributions

F.S. conceived the ideal, did calculations, and made simulations. F.S. and S.L.H. wrote the article and reviewed it together. S.L.H. supervised this study and finalized the manuscript.

## References

[b1] Pendry J. B., Schurig D. & Smith D. R. Controlling electromagnetic fields. Science 312, 1780–1782 (2006). 16728597 10.1126/science.1125907

[b2] Leonhardt U. Optical Conformal Mapping. Science 312, 1777–1780 (2006). 16728596 10.1126/science.1126493

[b3] Chen H. Y., Chan C. T. & Sheng P. Transformation optics and metamaterials. Nat. Mater. 9, 387–396 (2010). 20414221 10.1038/nmat2743

[b4] Leonhardt U. & Philbin T. G. Geometry and Light: The Science of Invisibility (Dover, 2010).

[b5] Xu Y., Du S., Gao L. & Chen H. Overlapped illusion optics: a perfect lens brings a brighter feature. New J. Phys. 13, 023010 (2011).

[b6] Zhang P. Theory of Transformation Optics and Invisibility Cloak Design. PhD. dissertation. KTH Royal Institute of Technology, Stockholm, Sweden (2011).

[b7] Jiang W. X., Qiu C.-W., Han T. C., Zhang S. & Cui T. J. Creation of Ghost Illusions Using Wave Dynamics in Metamaterials. Adv. Funct. Mater. 23, 4028–4034 (2013).

[b8] Yang F., Mei Z. L., Jin T. Y. & Cui T. J. DC electric invisibility cloak. Phys. Rev. Lett. 109, 053902 (2012). 23006174 10.1103/PhysRevLett.109.053902

[b9] Jiang W. X., Luo C. Y., Ma H. F., Mei Z. L. & Cui T. J. Enhancement of Current Density by dc Electric Concentrator. Sci. Rep. 2, 956; 10.1038/srep00956 (1012). 23233875 10.1038/srep00956 PMC3518816

[b10] Navau C., Prat-Camps J. & Sanchez A. Magnetic Energy Harvesting and Concentration at a Distance by Transformation Optics. Phys. Rev. Lett. 109, 263903 (2012). 23368564 10.1103/PhysRevLett.109.263903

[b11] Sun F. & He S. Create a uniform static magnetic field over 50T in a large free space region. Prog. Electromagn. Res. 137, 149–157 (2013).

[b12] Sun F. & He S. “DC magnetic concentrator and omnidirectional cascaded cloak by using only one or two homogeneous anisotropic materials of positive permeability.”. Prog. Electromagn. Res. 142, 683–699, 2013.

[b13] Sun F. & He S. Novel magnetic lens for static magnetic field enhancement. Progress In Electromagnetics Research Symposium Proceedings. Stockholm, Sweden, 1689–1691 (2013).

[b14] Liu K., Jiang W., Sun F. & He S. Experimental realization of strong DC magnetic enhancement with transformation optics. Prog. Electromagn. Res. 146, 187–194 (2014).

[b15] Sun F. & He S. Transformation inside a null-space region and a DC magnetic funnel for achieving an enhanced magnetic flux with a large gradient. Prog. Electromagn. Res. 146, 143–153 (2014).

[b16] Sun F. & He S. Static magnetic field concentration and enhancement using magnetic materials with positive permeability. Prog. Electromagn. Res. 142, 579–590 (2013).

[b17] Supradeep N. & Sato Y. DC Magnetic Cloak. Adv. Mater. 24, 71–74 (2012). 22114004 10.1002/adma.201104012

[b18] Gömöry F. *et al.* Experimental realization of a magnetic cloak. Science 335, 1466–1468 (2012). 22442477 10.1126/science.1218316

[b19] Wood B. & Pendry J. B. Metamaterials at zero frequency. J. Phys. Condens. Matter 19, 076208 (2007). 22251595 10.1088/0953-8984/19/7/076208

[b20] Wang R., Mei Z. L. & Cui T. J. A carpet cloak for static magnetic field. Appl. Phys. Lett. 102, 213501 (2013).

[b21] Aubry A. *et al.* Plasmonic light-harvesting devices over the whole visible spectrum. Nano Lett. 10, 2754 (2010). 10.1021/nl101235d 20518545

[b22] Lei D. Y., Aubry A., Maier S. A. & Pendry J. B. Broadband nano-focusing of light using kissing nanowires. New J. Phys. 12, 093030 (2010).

[b23] Lei D. Y., Aubry A., Luo Y., Maier S. A. & Pendry J. B. Plasmonic interactions between overlapping nanowires. ACS Nano 5, 597 (2011). 21190374 10.1021/nn102819p

[b24] Zhang S., Xia C. & Fang N. Broadband acoustic cloak for ultrasound waves. Phys. Rev. Lett. 106, 024301 (2011). 21405230 10.1103/PhysRevLett.106.024301

[b25] Chen H. & Chan C. T. Acoustic cloaking and transformation acoustics. J. Phys. D: Appl. Phys. 43, 113001 (2010).

[b26] Guenneau S., Amra C. & Veynante D. Transformation thermodynamics: cloaking and concentrating heat flux. Opt. Expr. 20, 8207–8218 (2012). 10.1364/OE.20.008207 22453491

[b27] Ma Y., Lan L., Jiang W., Sun F. & He S. A transient thermal cloak experimentally realized through a rescaled diffusion equation with anisotropic thermal diffusivity. NPG Asia Materials 5, e73 (2013).

[b28] Xu H., Shi X., Gao F., Sun H. & Zhang B. Ultrathin Three-Dimensional Thermal Cloak. Phys. Rev. Lett. 112, 054301 (2014). 24580599 10.1103/PhysRevLett.112.054301

[b29] Han T., Bai X., Thong J. T. L., Li B. & Qiu C.-W. Bilayer Isotropic Thermal Cloak. arXiv., 1307.2507 (2013).

[b30] Han T. C., Bai X., Thong J. T. L., Li B. & Qiu C.-W. Full Control and Manipulation of Heat Signatures: Cloaking, Camouflage and Thermal Metamaterials. Adv. Mater. 26, 1731–1734 (2014). 24497430 10.1002/adma.201304448

[b31] Narayana S. & Sato Y. Heat flux manipulation with engineered thermal materials. Phys. Rev. Lett. 108, 214303 (2012). 23003263 10.1103/PhysRevLett.108.214303

[b32] Wei Z., Cao Y., Han J., Wu C., Fan Y. & Li H. Broadband negative refraction in stacked fishnet metamaterial. Appl. Phys. Lett. 97, 141901 (2010).

